# Synaptic Plasticity as a Mechanism of Opioid Tolerance and Hyperalgesia

**DOI:** 10.3390/biology15080640

**Published:** 2026-04-18

**Authors:** Fenfen Qin, Qisheng Wang, Salahadin Abdi, Lingyong Li

**Affiliations:** 1 Department of Anesthesiology and Perioperative Medicine, University of Alabama at Birmingham, Birmingham, AL 35294, USA; 2 Department of Pain Medicine, The University of Texas MD Anderson Cancer Center, Houston, TX 77030, USA

**Keywords:** opioid analgesic tolerance, opioid-induced hyperalgesia, μ-opioid receptor, synaptic functional plasticity, synaptic structural plasticity, long-term potentiation

## Abstract

Opioid analgesics are essential in the management of severe and chronic pain; however, their prolonged use is limited by the onset of analgesic tolerance and opioid-induced hyperalgesia (OIH). Recent studies increasingly implicate both synaptic functional and structural plasticity within nociceptive pathways as crucial mechanisms in OIH and tolerance. This review integrates current mechanistic understanding of how opioids alter synaptic transmission throughout the dorsal root ganglia (DRG), spinal dorsal horn, and supraspinal nociceptive networks. Peripherally, μ-opioid receptor (MOR) activation on TRPV1-positive nociceptors initiates presynaptic long-term potentiation (LTP), forming an early substrate for central sensitization. In the spinal dorsal horn, chronic opioid exposure drives NMDAR-dependent LTP, TRPC-mediated calcium influx, and actin cytoskeleton remodeling, leading to persistent increases in synaptic strength and excitatory connectivity. In supraspinal regions—including the ventral hippocampus, prefrontal cortex, and amygdala—opioids promote experience-dependent plasticity and predictive coding, which link environmental cues to reduced analgesic effectiveness. In addition to synaptic functional plasticity, opioid-induced synaptic structural plasticity within nociceptive pathways has been shown to underlie the long-term nature of opioid analgesic tolerance. Collectively, these data define a distributed network of opioid-responsive synapses whose pathological potentiation underpins the development of tolerance and hyperalgesia. Elucidating these mechanisms underlying OIH and tolerance paves the way for targeted therapeutic strategies that maintain analgesic efficacy while minimizing adverse synaptic remodeling and negative outcomes.

## Simple Summary

Opioids are strong pain medicines that help many patients with serious pain. But when taken for a long time, the body gets used to the drugs and needs higher doses to get the same relief (this is called tolerance). Sometimes, these medicines can even make pain worse instead of better. This is called opioid-induced hyperalgesia. Here, we review the recent progress on why patients end up needing more opioids and why some feel more pain instead of less when taking opioid medications. In the spinal cord, long-term opioid use strengthens pain signals and changes the structure of nerve connections. In the brain, opioids affect areas that handle emotions and memories, so pain can feel worse or be linked to specific places or feelings. Because of these changes, patients may find that opioids stop working for them, or they feel more pain. Understanding how opioids affect the nervous system helps researchers and doctors find safer ways to manage pain, so patients can get relief without these unwanted effects.

## 1. Introduction of Opioid Analgesic Tolerance

Opioid analgesics are essential for managing moderate to severe pain, particularly in perioperative and cancer settings [1]. However, their clinical utility is limited by two adaptive responses that emerge with repeated use. Analgesic tolerance refers to a reduced analgesic effect that requires escalating doses, whereas opioid-induced hyperalgesia (OIH) describes a paradoxical increase in pain sensitivity caused by opioid exposure itself [2,3]. While these phenomena are mechanistically distinct, they often coexist, complicating clinical management. Tolerance is typically inferred when higher opioid doses restore analgesia, whereas OIH is suggested when pain intensifies or spreads despite dose escalation. This interplay can create a self-reinforcing cycle of escalating doses, thereby elevating the risk of dependence, respiratory depression, and opioid-related morbidity and mortality [2].

Opioid tolerance was historically regarded as a pharmacological phenomenon driven by μ-opioid receptor (MOR) desensitization, phosphorylation, and internalization. Early models focused on GPCR kinase (GRK)-dependent phosphorylation, β-arrestin-2 recruitment, and receptor endocytosis as the main determinants of tolerance [4,5]. However, growing clinical and preclinical evidence indicates that these receptor-level mechanisms alone do not fully explain the complexity, rapid onset, or persistence of tolerance and OIH [1]. Opioids activate an extensive network intracellular signaling pathways—including PKA, PKC, JNK, Src kinase, mTORC1, and CaMKII—that reshape neuronal excitability and synaptic signaling across the nociceptive system [6,7]. These pathways not only regulate MOR function but also initiate pronociceptive processes, such as NMDA receptor sensitization, TRPV1 channel facilitation, and microglial-mediated neuroinflammation, collectively shifting the nervous system toward a hyperexcitable state [8].

In addition to molecular signaling, opioids induce long-lasting forms of synaptic plasticity that resemble mechanisms of learning and memory processes [9]. This perspective reframes opioid tolerance as a systems-level plasticity disorder rather than merely a result of receptor desensitization. Opioid-induced plasticity manifests at various anatomical sites along the nociceptive neuroaxis [10]. In the periphery, primary afferent nociceptors in the dorsal root ganglia (DRG), notably TRPV1-positive C-fibers, undergo paradoxical presynaptic long-term potentiation (LTP), characterized by increased glutamate release following opioid washout [11]. In the spinal dorsal horn, prolonged opioid exposure promotes NMDAR-dependent postsynaptic potentiation, dendritic spine remodeling, and neuroimmune sensitization. These adaptations are especially prominent in lamina I and II circuits that integrate nociceptive input. These spinal adaptations intensify ascending nociceptive signaling and play a direct role in the development of OIH.

At the supraspinal level, opioids reshape circuits that integrate nociceptive, contextual, and affective information. These changes allow the brain to generate predictions about opioid availability and analgesic outcomes. Recent work shows that associative opioid tolerance is not distributed uniformly across the brain. Instead, it is mediated by a hierarchical circuit linking the ventral hippocampus (vHPC), dorsomedial prefrontal cortex (dmPFC), and basolateral amygdala (BLA) [12]. In this circuit, contextual cues stimulate glutamatergic vHPC inputs to dmPFC cholecystokinin-expressing (CCK^+^) neurons. These neurons subsequently project to the BLA, where they release CCK. CCK acts to suppress μ-opioid receptor signaling in BLA glutamatergic neurons, thereby diminishing morphine analgesia when the contexts have been previously associated with opioid administration. These findings indicate that supraspinal tolerance can arise through associative learning. Environmental cues activate a vHPC → dmPFC → BLA pathway that encodes predictive signals capable of reducing opioid analgesic efficacy [12].

Together, these findings support a contemporary view of tolerance and OIH as emergent consequences of maladaptive synaptic plasticity across peripheral, spinal, and supraspinal circuits. Opioids fundamentally alter synaptic function and structure throughout the nociceptive system. Circuits that normally mediate analgesia can be transformed into contributors to sensitization. This evolving framework prompts closer investigation of the specific functional and structural forms of synaptic plasticity engaged by opioids. Clarifying how opioids modify synaptic strength, receptor composition, ion channel activity, and dendritic morphology is essential for understanding tolerance and OIH. These insights will also help identify therapeutic strategies to counteract maladaptive plasticity.

Although substantial evidence supports synaptic plasticity as a major contributor to opioid tolerance and OIH, several mechanisms remain incompletely resolved or context-dependent. For example, presynaptic NMDARs on primary afferent terminals have been demonstrated in multiple preparations, yet their functional contribution varies across species, injury states, and experimental conditions. Likewise, opioid-induced LTP provides a compelling unifying framework, but its generalizability and causal relationship to behavioral tolerance remain debated. These uncertainties highlight the importance of distinguishing well-supported mechanisms—such as spinal NMDAR-dependent sensitization and microglial activation—from emerging pathways, including Tiam1–Rac1–mediated structural remodeling and TET1-dependent epigenetic regulation. Incorporating this distinction is essential for framing opioid tolerance not as a single canonical mechanism but as a constellation of interacting plasticity processes with varying levels of empirical support.

## 2. Synaptic Plasticity

Synaptic plasticity is defined as the ability of synapses to undergo activity-dependent modifications in strength, structure, and connectivity. Although traditionally regarded as the cellular foundation for learning and memory, these mechanisms are also exploited by chronic opioid exposure, leading to maladaptive reorganization of nociceptive circuits that gradually reduce analgesic efficacy and facilitate the development of OIH [13].

In this context, opioid-induced plasticity is generally classified into two interrelated categories. Functional plasticity alters synaptic transmission efficiency, whereas structural plasticity modifies synaptic architecture. These forms of plasticity are seldom independent; rather, they operate in concert to recalibrate nociceptive pathways throughout the nervous system. Elucidating how opioids disrupt these fundamental processes provides a mechanistic basis for understanding the emergence of tolerance, the onset of OIH, and the persistence of these adaptations even after opioid discontinuation.

### 2.1. Synaptic Functional Plasticity

Synaptic functional plasticity represents one of the earliest and most dynamic outcomes of opioid exposure, converting molecular signaling events into quantifiable alterations in synaptic efficacy [14]. With repeated activation of nociceptive circuits by opioids, the balance between excitatory and inhibitory transmission is recalibrated via changes in neurotransmitter release, receptor composition, and postsynaptic responsiveness. Although these adaptations resemble established mechanisms underlying learning and memory, chronic opioid exposure transforms them into maladaptive processes that intensify nociceptive drive instead of attenuating it [15].

A central aspect of this process is presynaptic LTP in primary afferent nociceptors, especially TRPV1-positive C-fibers. Instead of causing sustained inhibition, repeated or high-concentration MOR activation can paradoxically prime these terminals for increased glutamate release following opioid withdrawal—a phenomenon initially observed in spinal cord preparations [11,16]. This presynaptic potentiation is mediated by enhanced intracellular Ca^2+^ influx and second messenger signaling, leading to elevated miniature excitatory postsynaptic current (EPSC) frequency and greater paired-pulse depression, both indicative of increased presynaptic release probability.

On the postsynaptic side, chronic opioid exposure induces substantial receptor remodeling. NMDA and AMPA receptor subunits become upregulated, more heavily phosphorylated, and redistributed in ways that enhance postsynaptic excitability [8]. These receptor modifications are accompanied by ion channel remodeling, including changes in the expression and function of voltage-gated calcium, sodium, and potassium channels, which further sensitize dorsal horn neurons to nociceptive input [17]. These functional changes help explain several clinical features of opioid tolerance and OIH. Enhanced excitatory transmission contributes to increased pain sensitivity during ongoing opioid administration, particularly with short-acting agents such as remifentanil [18]. By enhancing nociceptive drive at early stages of sensory processing, synaptic functional plasticity establishes a feed-forward loop that hastens the progression from effective analgesia to tolerance and OIH, thereby complicating both perioperative pain management and long-term opioid therapy.

### 2.2. Synaptic Structural Plasticity

As functional adaptations accumulate, opioids also trigger a slower but more persistent form of plasticity: structural remodeling of synaptic architecture. Functional plasticity alters neurotransmission on rapid timescales, whereas structural plasticity consolidates these changes by reshaping the physical organization of synaptic connections [19]. This process involves modifications in dendritic spine density, morphology, and stabilization, all governed by actin cytoskeletal dynamics and small GTPase signaling.

Substantial evidence indicates that persistent nociceptive activity drives significant structural remodeling in spinal dorsal horn neurons. Our recent findings demonstrate that nerve injury leads to a marked increase in dendritic spine density and an elevated F-actin/G-actin ratio, with enhanced actin polymerization. Notably, these structural changes are abolished in Tiam1-deficient mice, implicating the Tiam1–Rac1 signaling pathway as a key regulator of nerve injury–induced structural plasticity [20]. Furthermore, accumulating research suggests that similar molecular mechanisms underlie morphine tolerance/OIH and neuropathic pain across the DRG nociceptors, spinal dorsal horn, and brain [21,22,23,24,25]. Consistently, our subsequent study found that morphine exposure increases dendritic spine density in spinal dorsal horn neurons via Tiam1–Rac1 signaling-dependent actin reorganization [26].

These structural modifications stabilize excitatory synapses and consolidate the functional changes underlying tolerance and OIH. By reshaping the connectivity of nociceptive circuits, structural plasticity facilitates the transition from acute opioid analgesia to chronic maladaptive sensitization. Synaptic functional and structural plasticity together establish a synergistic framework that enables opioids to remodel pain-processing circuits. These neuroadaptations manifest at various levels of the nociceptive system, including the DRG and spinal dorsal horn, collectively contributing to the development of opioid tolerance and hyperalgesia. Gaining a deeper understanding of the interaction between these forms of plasticity is critical for identifying therapeutic strategies that disrupt maladaptive opioid responses while maintaining analgesic effectiveness.

## 3. Synaptic Plasticity in the DRG and Spinal Cord for Opioid Analgesic Tolerance

Opioid analgesic tolerance arises from a complex interplay of presynaptic and postsynaptic plasticity mechanisms distributed throughout nociceptive pathways. The DRG and spinal dorsal horn, as the primary central relay points for nociceptive transmission, serve as critical sites where opioids produce enduring maladaptive changes. Substantial evidence indicates that both repeated and brief exposure to MOR agonists trigger a series of synaptic modifications in primary afferent terminals and dorsal horn neurons, leading to reduced opioid efficacy and the development of OIH [8]. These adaptations include presynaptic LTP in DRG afferents, postsynaptic LTP, and structural remodeling within spinal neurons, collectively forming a distributed network of plasticity that underlies tolerance.

### 3.1. Presynaptic Plasticity in DRG Neurons for Opioid Analgesic Tolerance

Presynaptic plasticity in DRG neurons is triggered by sustained nociceptive activity, which recruits both protein kinase C (PKC) and Src family kinases (SFKs)—two signaling pathways converging on NMDAR regulation. PKC phosphorylates the intracellular domains of GluN2 subunits, enhancing local depolarization and thereby reducing the voltage dependence of the Mg^2+^ block [27,28]. Concurrently, SFKs—particularly Fyn—promote high-conductance NMDAR states through tyrosine phosphorylation of GluN2B, a mechanism well characterized in spinal nociceptive circuits [29,30]. Collectively, these kinase pathways constitute a “two-key” mechanism that permits presynaptic NMDAR activation at subthreshold membrane potentials, a phenomenon first demonstrated in primary afferent terminals [31]. Once this permissive, kinase-sensitized state is established, α2δ-1 acts as a critical amplifier of presynaptic NMDAR function. α2δ-1 forms a stable complex with GluN1/GluN2 subunits, promoting increased receptor surface trafficking and synaptic retention [32,33]. In DRG neurons, injury- or opioid-induced upregulation of α2δ-1 substantially enhances presynaptic NMDAR-mediated currents and Ca^2+^ influx [16]. This structural stabilization not only increases the number of NMDARs available for activation but also favors high-open-probability conformations, thereby amplifying presynaptic glutamate release. Consistently, genetic or pharmacological disruption of α2δ-1 reduces neuropathic pain and opioid-induced hyperalgesia, highlighting its essential role in pathological presynaptic sensitization [8,10].

**Figure 1:**
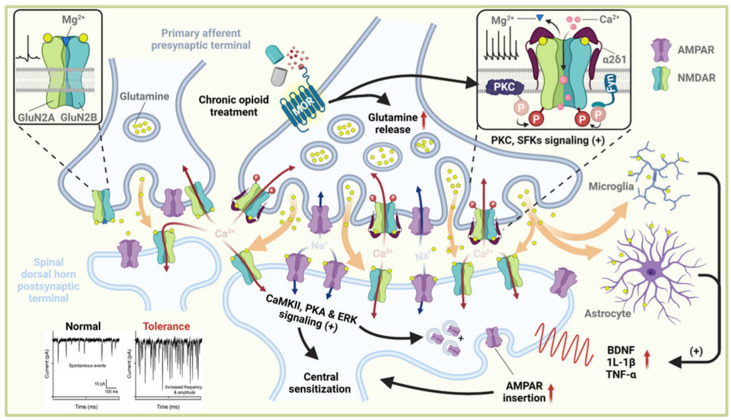
Coordinated presynaptic and postsynaptic plasticity mechanisms driving opioid tolerance and OIH. Chronic opioid exposure induces maladaptive synaptic plasticity at the first nociceptive synapse between DRG afferents and spinal dorsal horn neurons. Presynaptic NMDAR sensitization. PKC and SFKs phosphorylate presynaptic NMDARs, lowering their activation threshold. The auxiliary subunit α_2_δ-1 stabilizes NMDAR surface expression and enhances Ca^2+^ permeability. These changes promote Ca^2+^ influx and increase glutamate release from DRG terminals. Excess glutamate diffuses across the cleft, activating postsynaptic receptors and glial cells. Epigenetic reprogramming enhances BDNF transcription and excitatory drive. Glial-derived cytokines and BDNF further amplify excitatory tone and reduce inhibitory input, resulting in circuit-level polarization. Integrated outcome. Presynaptic LTP and postsynaptic hyperexcitability form a feedforward loop that progressively diminishes opioid analgesic efficacy and promotes the development of opioid tolerance and OIH. Created in BioRender. Wang, Q. (2026) https://BioRender.com/xoyf7bw (accessed on 10 March 2026).

The resulting Ca^2+^ influx through presynaptic NMDARs directly increases vesicle release probability by elevating Ca^2+^ microdomains that recruit synaptotagmin, Munc13, and other Ca^2+^-dependent release machinery [34,35]. This mechanism is well established in primary afferent terminals, where NMDAR-mediated Ca^2+^ entry enhances both spontaneous and evoked glutamate release [34]. Accordingly, presynaptic NMDAR activation elevates the frequency of miniature excitatory postsynaptic currents (mEPSCs) and alters paired-pulse ratios, reflecting a substantial increase in release probability [36,37]. These presynaptic modifications significantly amplify excitatory input to dorsal horn neurons, providing a direct feedforward mechanism that drives NMDAR-dependent synaptic facilitation and contributes to the initial stages of spinal sensitization [38,39]. Increased glutamatergic output from DRG terminals sustains dorsal horn excitability, engaging both neuronal and glial components of central sensitization [40]. Elevated glutamate concentrations facilitate NMDAR-dependent wind-up and long-term potentiation in dorsal horn neurons, thereby strengthening excitatory synaptic transmission and lowering the activation threshold. Persistent activation of CaMKII, PKA, and ERK signaling further promotes AMPA receptor (AMPAR) insertion, dendritic remodeling, and increased membrane excitability [23,41]. In parallel, excessive glutamate release activates astrocytes and microglia, inducing the production of brain-derived neurotrophic factor (BDNF), interleukin-1β (IL-1β), and tumor necrosis factor-α (TNF-α), which further enhance excitatory drive and suppress inhibitory tone [42,43]. Collectively, these processes convert presynaptic NMDAR-driven facilitation into a maladaptive state of plasticity that undermines μ-opioid receptor–mediated analgesia and promotes the development of opioid tolerance (Figure 1).

### 3.2. Postsynaptic Plasticity in Spinal Dorsal Horn Neurons for Opioid Analgesic Tolerance

In parallel with presynaptic changes, chronic opioid exposure leads to substantial postsynaptic remodeling in spinal dorsal horn neurons, particularly in lamina I–II excitatory interneurons that integrate nociceptive input. These neurons exhibit increased excitability, reflected by heightened NMDA receptor activity, elevated intracellular Ca^2+^ signaling, and significant dendritic spine reorganization. Recent studies describe this process as a form of “neural circuitry polarization,” in which heightened excitatory drive and weakened inhibitory control shift dorsal horn microcircuits toward a persistently hyperresponsive state [44].

**Figure 2:**
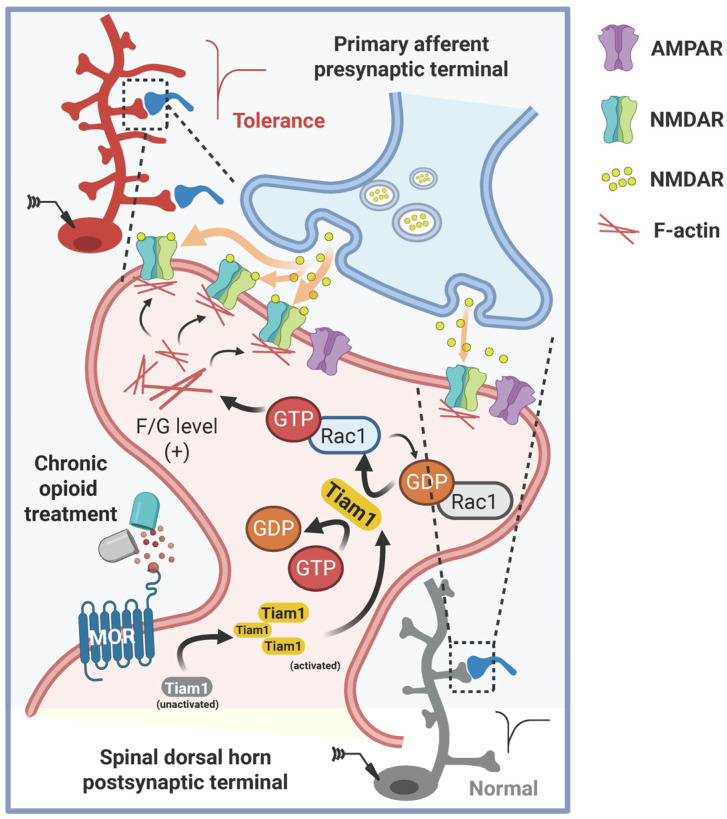
Tiam1-mediated synaptic functional and structural plasticity in spinal dorsal horn neurons underlying opioid tolerance and OIH. In spinal dorsal horn neurons, chronic opioids activate the Tiam1–Rac1 signaling, which is essential for the development and maintenance of opioid tolerance by orchestrating synaptic structural and functional plasticity via actin cytoskeleton reorganization and synaptic NMDAR stabilization. Created in BioRender. Wang, Q. (2026) https://BioRender.com/7us8nn5 (accessed on 10 March 2026).

Recent work has identified Tiam1, a Rac1-specific guanine nucleotide exchange factor, as a regulator of actin-dependent dendritic spine remodeling in spinal dorsal horn neurons. Chronic morphine exposure activates Tiam1–Rac1 signaling, promoting actin polymerization, increased spine density, and stabilization of excitatory synapses. These structural changes enhance postsynaptic excitability and facilitate NMDAR-dependent potentiation. Although these findings provide mechanistic insight into how opioids reshape postsynaptic architecture, current evidence is derived primarily from rodent models, and the broader relevance of Tiam1-mediated remodeling across pain states and species remains to be established. Thus, Tiam1–Rac1 signaling is viewed as an emerging contributor to opioid-induced structural plasticity rather than a universally conserved pathway (Figure 2) [20,26].

Epigenetic regulation also contributes to postsynaptic sensitization. Ten-eleven translocation methylcytosine dioxygenase 1 (TET1), a DNA demethylation enzyme, is upregulated in dorsal horn neurons under inflammatory and neuropathic conditions and promotes demethylation of genes such as BDNF, thereby enhancing excitatory synaptic transmission [45]. Inhibition of TET1 reduces aberrant excitatory drive and mitigates pain hypersensitivity, suggesting a role for TET1-dependent epigenetic remodeling in sustaining postsynaptic hyperexcitability [46]. However, the involvement of TET1 in opioid-specific tolerance remains less well defined, and its contribution likely represents one component of a broader epigenetic landscape. Accordingly, TET1 should be considered an emerging mechanism rather than a core determinant of opioid-induced plasticity.

Collectively, these mechanisms—structural remodeling, epigenetic enhancement of excitatory synapses, and metabolic regulation—converge to establish a persistently hyperexcitable postsynaptic state in the spinal dorsal horn. In conjunction with presynaptic LTP and increased glutamate release from DRG afferents, this postsynaptic sensitization creates a coordinated maladaptive network that steadily undermines opioid analgesia and accelerates the onset of opioid tolerance.

Neuroimmune signaling represents a major driver of opioid-induced hyperalgesia. Chronic opioid exposure activates spinal microglia through receptors such as P2X7 and TLR4, leading to the release of IL-1β, TNF-α, and BDNF, which enhance excitatory transmission and reduce inhibitory tone [47,48,49]. Although microglial MOR signaling was once proposed as a primary trigger, Corder et al. demonstrated that MORs are expressed on nociceptors—not microglia—and that deleting nociceptor MORs abolishes morphine tolerance and OIH [10]. These findings indicate that microglia are activated secondarily by nociceptor-driven plasticity, yet once engaged, they strongly amplify dorsal horn hyperexcitability. Thus, neuroimmune mechanisms should be considered integral components of the maladaptive plasticity underlying OIH, acting in parallel with presynaptic NMDAR sensitization and postsynaptic structural remodeling.

## 4. Synaptic Plasticity in the Brain for Opioid Analgesic Tolerance

The spinal mechanisms outlined above—presynaptic NMDAR sensitization, postsynaptic structural and epigenetic remodeling, and the development of a feedforward excitatory loop within the dorsal horn—constitute only the initial tier of a hierarchical neuroplastic cascade. As nociceptive signals ascend and engage supraspinal circuits, chronic opioid exposure triggers a secondary wave of plasticity that reorganizes brainstem, limbic, cortical, and reward pathways. These supraspinal adaptations not only intensify spinal sensitization but also embed opioid tolerance within neural systems governing affect, cognition, prediction, and reinforcement, thereby transforming opioid tolerance into a distributed, experience-dependent brain disorder [50,51].

Within the descending pain-modulatory system, chronic opioid administration reorganizes synaptic transmission in the periaqueductal gray (PAG) and rostral ventromedial medulla (RVM). Prolonged opioid exposure diminishes GABAergic inhibition in the PAG and shifts RVM output from analgesic OFF-cell predominance toward pronociceptive ON-cell facilitation—a form of opioid-induced metaplasticity that transforms analgesic circuits into facilitators of hyperalgesia [51]. Concurrent research demonstrates that NMDA receptor–dependent anti-opioid systems, particularly those involving NR2A-containing NMDARs, are upregulated in the PAG and ventral tegmental area (VTA) during chronic opioid exposure, thereby enhancing glutamatergic counter-regulation and accelerating the development of tolerance. Restoration of NR2A expression in the PAG or VTA, but not the nucleus accumbens, reinstates morphine tolerance in NR2A-deficient mice, providing evidence for locus-specific supraspinal plasticity [52].

Beyond brainstem circuits, chronic opioid administration induces profound neuroplasticity in limbic and cortical regions that govern the emotional, cognitive, and contextual dimensions of pain [53]. Chronic opioid exposure disrupts the excitatory–inhibitory balance, dendritic spine morphology, and synaptic strength in the ventral hippocampus (vHPC) [12], prefrontal cortex (mPFC) [54,55], anterior cingulate cortex (ACC) [56], and basolateral amygdala (BLA) [57,58]. These alterations contribute to heightened pain affect, impaired endogenous analgesia, and increased salience of pain-related cues, illustrating that opioid tolerance is not merely a pharmacological phenomenon but also a learned and context-dependent process. Electrophysiological studies further demonstrate that repeated morphine exposure disrupts LTP and paired-pulse dynamics across the hippocampal longitudinal axis, resulting in region-specific changes at Schaffer collateral–CA1, temporoammonic–CA1, and perforant pathway–dentate gyrus (DG) synapses [59]. Such modifications reflect a shift toward pathological LTP or impaired synaptic integration, mechanisms believed to underlie aberrant memory formation associated with opioid tolerance. Structural plasticity further consolidates these functional alterations. Conditioned morphine tolerance enhances adult hippocampal neurogenesis, increases dendritic spine density in CA1 and dentate gyrus (DG) neurons, and upregulates pro-plasticity molecules such as BDNF, TrkB, and RhoA in the ventral hippocampus [60]. These structural adaptations indicate that associative opioid tolerance engages hippocampal learning mechanisms, embedding opioid-related contextual memories within enduring neural circuitry.

This is consistent with the identification of a hierarchical vHPC → dmPFC → BLA circuit that encodes predictive associations between environmental cues and opioid availability. Chronic morphine administration increases excitability and glutamatergic output of dmPFC cholecystokinin-positive (CCK^+^) neurons, which release CCK in the BLA to attenuate μ-opioid receptor–mediated inhibition, thereby diminishing opioid analgesic efficacy in drug-associated contexts [12]. Disruption of any component of this circuit abolishes associative tolerance without impacting acute analgesia, illustrating that supraspinal plasticity can encode learned predictions that proactively reduce opioid analgesia.

Finally, opioids induce remodeling of reward circuitry within the nucleus accumbens (NAc), where D1- and D2-type medium spiny neurons (MSNs) undergo allostatic reorganization of dopamine–cyclic AMP (cAMP) signaling. While acute opioid exposure initially enhances D1-MSN responsiveness, chronic administration shifts the balance toward D2-MSN predominance, driven by changes in dopamine transporter expression and phosphodiesterase activity that alter cAMP kinetics [61]. This allostatic adaptation raises the reward threshold, reduces the hedonic value of opioids, and promotes escalating drug consumption—processes that are closely linked to the development of analgesic tolerance. Together, these supraspinal adaptations integrate descending facilitation, limbic–cortical remodeling, predictive coding, and reward allostasis into a cohesive systems-level framework that accounts for the persistence, generalization, and context dependence of opioid analgesic tolerance.

## 5. Conclusions

Opioid analgesic tolerance and opioid-induced hyperalgesia arise from a complex cascade of synaptic plasticity mechanisms spanning peripheral, spinal, and supraspinal nociceptive circuits. Peripherally, activation of μ-opioid receptors on TRPV1-positive nociceptors initiates presynaptic long-term potentiation, thereby promoting ascending central sensitization and establishing the initial substrate for maladaptive opioid responses. Within the spinal dorsal horn, both functional and structural forms of plasticity—including NMDAR-dependent potentiation, transient receptor potential canonical (TRPC)-mediated calcium signaling, and cytoskeleton-driven synaptic remodeling—consolidate these changes into persistent alterations in synaptic strength and connectivity. In supraspinal regions, particularly limbic and corticolimbic circuits, associative and contextual components of tolerance are encoded, integrating emotional, environmental, and learning-related factors into the chronic opioid response.

From a translational standpoint, these mechanistic insights also help explain why clinically used opioids differ in their propensity to induce tolerance and OIH. Short-acting opioids such as remifentanil produce rapid fluctuations in MOR occupancy, strongly engaging presynaptic LTP and rebound glutamate release, consistent with abrupt postoperative hyperalgesia [62]. High-efficacy opioids like fentanyl, with strong β-arrestin bias and efficient receptor internalization, preferentially recruit intracellular signaling cascades and neuroimmune activation [1]. In contrast, morphine’s slower internalization kinetics and stronger engagement of microglial TLR4/P2X7 pathways contribute to more pronounced glial-dependent hyperalgesia [63,64]. Partial agonists such as buprenorphine, which induce weaker NMDAR sensitization and minimal microglial activation, show a comparatively lower incidence of OIH [65]. These opioid-specific patterns of synaptic and neuroimmune plasticity provide a mechanistic basis for their distinct clinical profiles.

Several pathways highlighted in this review also represent actionable therapeutic targets. Presynaptic NMDARs, α_2_δ_1_-dependent synaptic stabilization, TRPC-mediated Ca^2+^ influx, and microglial P2X7/TLR4 signaling have all shown therapeutic potential in preclinical models of tolerance and OIH. In parallel, molecules such as BDNF, IL-1β, and epigenetic regulators including TET1 may serve as measurable biomarkers of opioid-evoked plasticity and central sensitization, potentially enabling mechanism-informed patient stratification. Linking these mechanistic signatures to opioid-specific clinical outcomes—such as dose-escalation trajectories, OIH incidence, and responses to adjuvant therapies—will be essential for translating basic discoveries into personalized pain management strategies.

## 6. Future Direction

Future research should elucidate the cell-type-specific architecture of opioid-induced plasticity within nociceptive circuits, identify molecular mechanisms that convert acute opioid inhibition into long-term potentiation, and develop targeted interventions that preserve analgesic efficacy while preventing maladaptive synaptic reorganization. Additional priorities include delineating the interactions between chronic pain states and opioid-evoked plasticity, as well as specifying sex- and genotype-dependent determinants of tolerance vulnerability. Establishing a comprehensive mechanistic framework that integrates these factors will be essential for advancing next-generation analgesics capable of sustaining efficacy without promoting tolerance or hyperalgesia.

## Data Availability

No new data were created or analyzed in this study. Data sharing is not applicable to this article.
